# Increased glutamic acid decarboxylase expression in the hypothalamic suprachiasmatic nucleus in depression

**DOI:** 10.1007/s00429-017-1442-y

**Published:** 2017-06-12

**Authors:** Xueyan Wu, Rawien Balesar, Jing Lu, Sahar Farajnia, Qiongbin Zhu, Manli Huang, Ai-Min Bao, Dick F. Swaab

**Affiliations:** 1 Department of Neurobiology, Key Laboratory of Medical Neurobiology of Ministry of Health of China, Zhejiang Province Key Laboratory of Neurobiology, Zhejiang Province Key Laboratory of Mental Disorder’s Management, National Clinical Research Center for Mental Health Disorders, Zhejiang University School of Medicine, 866 Yuhangtang Road, Hangzhou, 310058 Zhejiang People’s Republic of China; 2 Department of Human Anatomy, School of Basic Medical Sciences, Anhui Medical University, 81 MeiShan Road, Hefei, 320023 People’s Republic of China; 3 Netherlands Institute for Neuroscience, an Institute of the Royal Netherlands Academy of Arts and Sciences, University of Amsterdam, Meibergdreef 47, 1105 BA Amsterdam, The Netherlands; 4 Department of Mental Health, Zhejiang Province Key Laboratory of Mental Disorder’s Management, National Clinical Research Center for Mental Health Disorders, First Affiliated Hospital, Zhejiang University School of Medicine, 79 Qing Chun Road, Hangzhou, 310003 People’s Republic of China

**Keywords:** Depression, Suprachiasmatic nucleus, GABA, Arginine vasopressin, Sex difference

## Abstract

In depression, disrupted circadian rhythms reflect abnormalities in the central circadian pacemaker, the hypothalamic suprachiasmatic nucleus (SCN). Although many SCN neurons are said to be GABAergic, it was not yet known whether and how SCN GABA changes occur in the SCN in depression. We, therefore, studied GABA in the SCN in relation to the changes in arginine vasopressin (AVP), which is one of the major SCN output systems. Postmortem hypothalamus specimens of 13 subjects suffering from depression and of 13 well-matched controls were collected. Quantitative immunocytochemistry was used to analyze the protein levels of glutamic acid decarboxylase (GAD)65/67 and AVP, and quantitative in situ hybridization was used to measure transcript levels of GAD67 in the SCN. There were a significant 58% increase of SCN GAD65/67-ir and a significant 169% increase of SCN GAD67-mRNA in the depression group. In addition, there were a significant 253% increase of AVP-ir in female depression subjects but not in male depression patients. This sex difference was supported by a re-analysis of SCN AVP-ir data of a previous study of our group. Moreover, SCN-AVP-ir showed a significant negative correlation with age in the control group and in the male, but not in the female depression group. Given the crucial role of GABA in mediating SCN function, our finding of increased SCN GABA expression may significantly contribute to the disordered circadian rhythms in depression. The increased SCN AVP-ir in female—but not in male-depression patients—may reflect the higher vulnerability for depression in women.

**Electronic supplementary material:**

The online version of this article (doi:10.1007/s00429-017-1442-y) contains supplementary material, which is available to authorized users.

## Introduction

In mammals, the proper temporal organization of behavioral, physiological, and biochemical processes in synchronization with the environmental light/dark cycle is regulated by the central circadian pacemaker, the hypothalamic suprachiasmatic nucleus (SCN). In depression, these biological rhythms become disrupted. Fluctuating mood, such as early morning mood worsening, is a feature of major depressive disorder (MDD) (Morris et al. 2009), while in seasonal affective disorder (SAD), low mood onset and a relapse of depressive episodes begin when photoperiod shortens (Lewy et al. 2006). In addition, almost 90% of the MDD patients suffer from sleep disturbances (Neylan 1995), while light therapy, which acts via re-setting of the disrupted SCN, has proven to be a successful treatment not only for SAD patients (Martensson et al. 2015), but also for MDD patients (Yamada et al. 1995). Polymorphisms in SCN clock genes were also found to be a risk factor for bipolar disorder (BD) (Rybakowski et al. 2014). In our previous studies, we found a disturbance in the key output neuropeptide of the SCN, arginine vasopressin (AVP), in depression (Zhou et al. 2001).

A hyperactive hypothalamo–pituitary–adrenal (HPA) axis is often a feature of depression (Bao et al. 2008), and activation of the corticotropin-releasing hormone (CRH) neurons in the hypothalamic paraventricular nucleus (PVN) is the central motor for HPA activity (for review, see Bao et al. 2008). Direct and indirect projections from the SCN to the PVN have been found in mouse and rat (Abrahamson et al. 2001) as well as in human (Dai et al. 1998). In nocturnal animals, such as rats, SCN AVP was found to inhibit corticosterone release (Kalsbeek et al. 1996). However, in a diurnal animal, the A. ansorgei, SCN AVP was found to stimulate corticosterone release (Kalsbeek et al. 2008). Most SCN neurons in rat and human are reported to be GABAergic (producing γ-aminobutyric acid as a neurotransmitter), and contain glutamic acid decarboxylase (GAD), which is the key enzyme for GABA production (Moore and Speh 1993; Buijs et al. 1995; Gao and Moore 1996). In rodents, the SCN consists of two parts: the ‘core’, which comprises vasoactive intestinal peptide (VIP) and gastrin-releasing peptide-producing neurons, and the ‘shell’, which mainly contains AVP-producing neurons (Moore et al. 2002). GABA is essential for interregional communication within the SCN network and can act both as an excitatory and inhibitory neurotransmitter in an adult rodent’s SCN (De Jeu and Pennartz 2002; Choi et al. 2008; Albus et al. 2005; Irwin and Allen 2009). This difference is probably due to the differential expression of the chloride cotransporters KCC1 and NKCC2 in the SCN (Choi et al. 2008; Belenky et al. 2010). It seems that GABAergic excitation relays the photic and phase information from ventral to dorsal SCN following a shifted light/dark cycle (Albus et al. 2005). Increased SCN GAD65-mRNA was observed in rats exposed to chronic intermittent stress (Bowers et al. 1998). These findings suggest that disordered GABA expression contributes to the disturbance of SCN functions in depression, in a way that may be distinct from rodents, which are nocturnal animals. To test this hypothesis, we used immunocytochemistry to measure the amount of GAD65/67-immunoreactivity (ir) and AVP-ir in the SCN, and used in situ hybridization to quantify the expression of SCN GAD67-mRNA, which is the dominant GAD-mRNA type in the SCN (Gao and Moore 1996), from a series of human hypothalamic tissue samples from individuals with a long-term history of depression as well as from well-matched controls. Since immunoreactive GAD fibers may arise from both inside and outside the SCN, special attention was paid to the quantification of the GAD mRNA. In addition, special attention was paid to potential sex differences, since depression is more prevalent in women than in men (Altemus 2006).

## Methods

### Brain material

Hypothalamic material was obtained from The Netherlands Brain Bank following permission for a brain autopsy and the use of brain material and clinical data for research purposes. The diagnosis of MDD or BD at any time during life was made by qualified psychiatrists according to the Diagnostic and Statistical Manual of Mental Disorders (DSM)-III-R/DSM-IV criteria. The absence of neuropathological changes, both in the depression group and in the control group, was confirmed by systematic neuropathological investigation. In total, 13 depressed patients (6 MDD, 7 BD) and 13 control subjects were studied. To control for experimental variance and to reduce biological variance between groups, the MDD and BD groups (MDD + BD), both separately and pooled, were matched with their respective controls for sex, age, post-mortem delay, season and clock time of death, brain weight, cerebrospinal fluid-pH, and fixation time. Detailed clinico-pathological information and *P* values of the parameter-matching are given in the table in Supplement.

### Immunocytochemistry of GAD65/67-ir and AVP-ir in the SCN

The hypothalami were dissected at autopsy and fixed in 0.1 M phosphate buffered 4% w/v formaldehyde (pH 7.2) for 1–2 months. Tissues were dehydrated in graded ethanol, embedded in paraffin, and serially cut into sections (6 μm) on a Leitz microtome and stored at room temperature (RT). The borders of the SCN were determined at the level of the optic chiasm by staining AVP in every 50th section. The rostral and caudal SCN borders were defined as the sections in which no AVP cells were present. For each AVP-stained section, an adjacent section was stained by GAD65/67.

The specificity of the antibody of the mouse monoclonal anti-AVP (D-7, a gift from Dr. A. Silverman to Dr. F. W. Van Leeuwen, The Netherlands Institute for Brain Research, Amsterdam, The Netherlands) was determined earlier, and the same antibody was successfully applied to the human brain tissues in a previous study of our group (Wu et al. 2006). We confirmed that this AVP antibody does not cross-react with oxytocin in the staining protocol used in the present study (see Supplement Figure 1). The specificity of the polyclonal rabbit anti-GAD65/67 antibody (Chemicon Int. Temecula, CA, USA. AB1511) has also been determined previously by us and other groups (Gao et al. 2013). AVP and GAD65/67 staining protocols have been described, see details in Methods in Supplement.

### In situ hybridization for GAD67-mRNA in the SCN

**Fig. 1:**
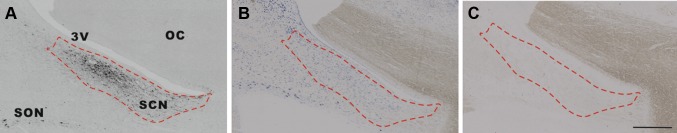
Specific in situ hybridization signals of glutamic acid decarboxylase (GAD)67-mRNA in the suprachiasmatic nucleus (SCN). Delineation of the SCN by arginine vasopressin (AVP) immunocytochemical staining (**a**). In adjacent sections (**b**, **c**), a specific GAD67-mRNA in situ hybridization signal (**b**) was observed in the SCN, as shown by the *dashed line* by the GAD67 antisense probe, while no signal was found in the entire section (**c**) by a scrambled probe. *3V* the third ventricle, *OC* optic chiasm, *SON* supraoptic nucleus. *Scale bar* 0.5 mm

The locked nucleic acid (LNA)-modified probes for GAD67-mRNA were obtained from Roche, Germany with FAM denoting a fluorescein tag, an LNA at every third position, and ma 2′*O*-methyl modified ribonucleic acid. Two antisense probes (probe 1 and probe 2) were available, both of which were complementary to human GAD67. The sequence of probe 1 was: 5′ AAG CTG GTT GGC AGC ATG T 3′, complementary to bases 2548–2566 of human GAD67 (GeneBankNM_00817). The sequence of probe 2 was: 5′ AAG GUG TCU CUG CAG TCA A 3′, complementary to bases 931–949 of human GAD67 (GeneBankNM_000817.2). We observed a similar GAD67-mRNA distribution with both probes (data not shown) and we chose probe 1 to quantify GAD67-mRNA expression in the present study. In addition, a scrambled probe was used as control, with a sequence of 5′ TGG TCU ACG TAU GCC AUG T 3′. The specificity of probe 1 is shown in Fig. 1.

**Fig. 2:**
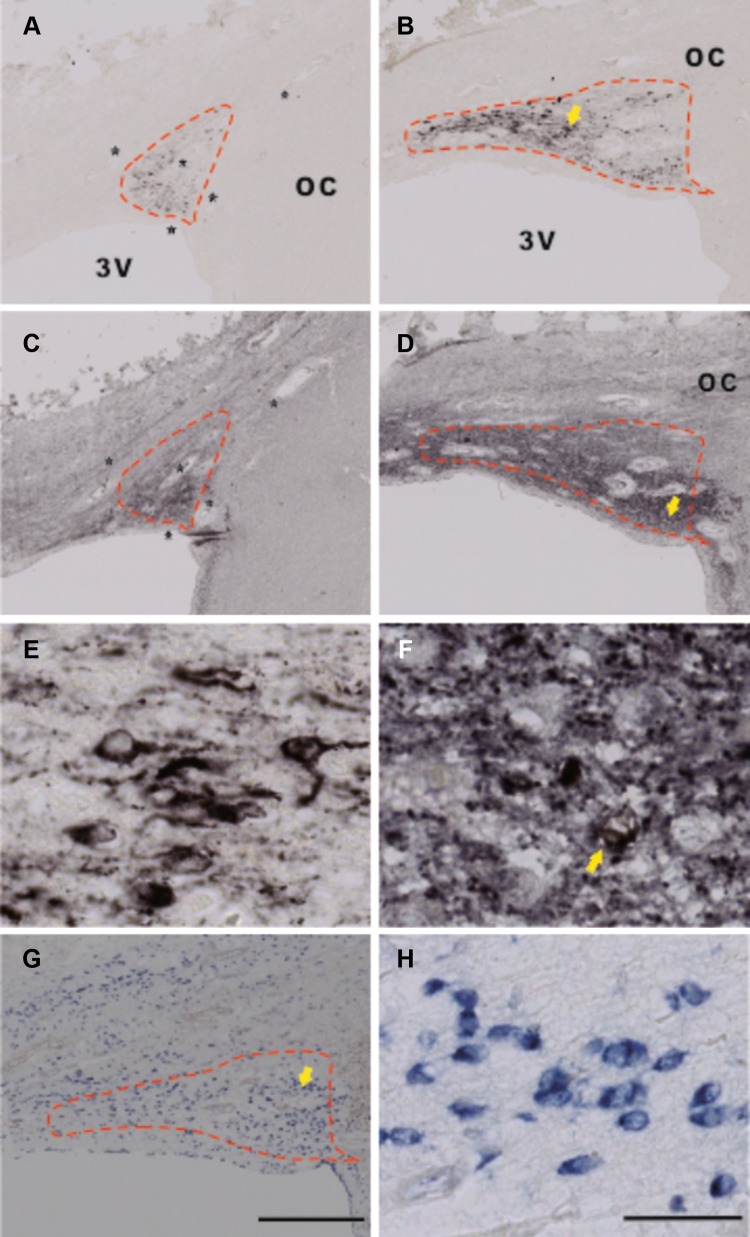
Immunocytochemical staining of arginine vasopressin (AVP), glutamic acid decarboxylase (GAD)65/67, and in situ hybridization (ISH) of GAD67-mRNA in the suprachiasmatic nucleus (SCN). **a**, **b**, **e** AVP immunoreactivity (ir), **c**, **d**, **f** GAD65/67-ir, and **g**, **h** GAD67-mRNA. C is an adjacent section to **a**, while **d** is an adjacent section to **b**, respectively. **e**, **f**, **h** Higher magnification of **b**, **d,** and **g**, respectively, from the area indicated by an *arrow*. Please note that AVP-staining is mainly present in neurons, but also in fibers, providing a clear boundary for the SCN (**a**, **b**, **e**), while GAD65/67-staining is largely present in SCN fibers (**c**, **d**, **f**) and only in a few of its neurons in some patients (indicated by an *arrow* in **f**). The SCN area was outlined according to an AVP-stained section (as shown by the *dashed line*), and the outline was transferred to the adjacent section with GAD65/67-staining or GAD67-mRNA ISH, based upon at least four markers in the area, as indicated by *stars* (usually blood vessels) in **a** and **c**. Please also note that almost all SCN neurons were surrounded by GAD65/67-ir beads (**f**), which probably represent synapses. In the rostral SCN, GAD-ir outlined the SCN (**c**) in almost the same way as AVP-ir (**a**), but in the more caudal part of the SCN, GAD65/67-staining was also distributed over a larger hypothalamic area around the SCN except for the optic tract and optic chiasm (OC) (**d**). GAD67-mRNA was abundantly distributed over the SCN and its surroundings (**g**). *3V* third ventricle. *Scale bar* in **a**–**d**, **g** 0.5 mm, **e**, **f**, **h** 0.05 mm

In situ hybridization for GAD67-mRNA was performed in every 100th section within the SCN area delineated by the adjacent AVP-staining section (Fig. 2) with the protocol we previously described (Zhu et al. 2016), see Methods in Supplement.

### Image analysis

Quantitative image analyses were carried out by one investigator (X-Y Wu), who was blind to the diagnosis and experimental conditions. Quantification of the integrated optical density (IOD) of immunocytochemistry or in situ hybridization signals was performed as described in our previous studies (Zhu et al. 2016). The setup consisted of an image analysis system (Image Proversion 6.3, Media Cybernetics, Rockville, USA) connected to a black and white camera (SONY XC-77E) mounted on a microscope (Zeis Axios-kop with Plan-NEOFLUAR Zeiss objectives, Carl Zeiss GmbH, Jena, Germany). The SCN area covered by AVP-stained neurons was outlined manually at a 20× objective. The outline of the SCN was then transferred to the adjacent GAD65/67-ir or GAD67-mRNA stained image by selecting at least four corresponding marker points in both images (see Fig. 2a, c). For data collection and calculation, see Methods in Supplement.

### Statistical analysis

Since the data were not always normally distributed, nonparametric statistics were applied for data analysis. All data are given as median (25th–75th percentile). The differences between two groups were evaluated by Mann–Whitney *U* test. Differences among more than two groups were first evaluated by means of the Kruskal–Wallis test and, if significant, were further evaluated by means of the Mann–Whitney *U* test between groups. A multiple linear regression model was also applied to confirm whether AVP-ir was indeed associated with sex, taking into consideration confounding factors, such as age, depression, postmortem delay, fixation time, and brain weight. Correlations were examined with the Spearman test. Differences in clock time and month of death were analyzed with the Mardia–Watson–Wheeler test. All tests were two-tailed and *P* values <0.05 were considered to be significant. SPSS 23.0 was applied for the data analysis.

## Results

**Fig. 3:**
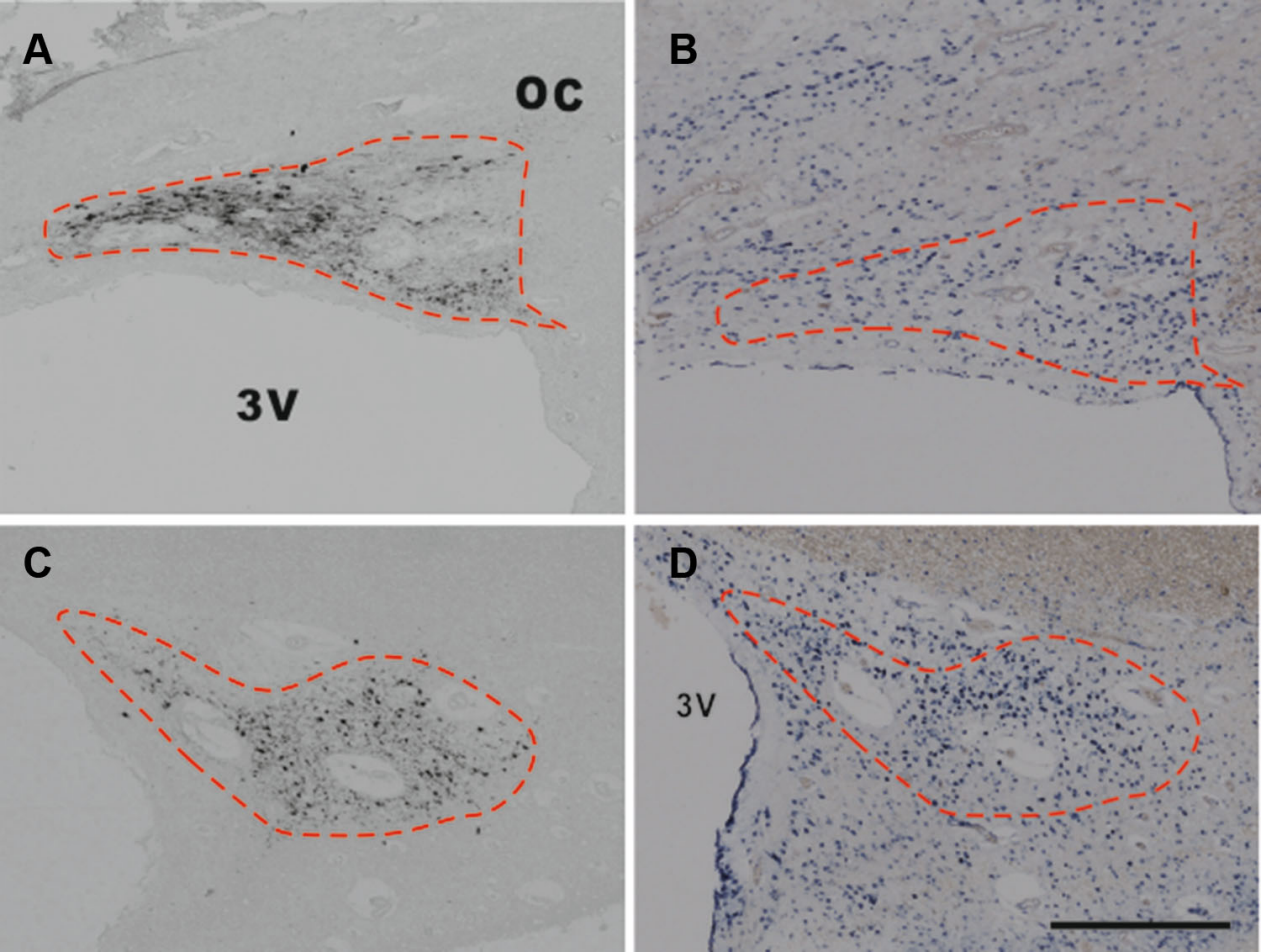
Representative distributions of the immunocytochemical signals of arginine vasopressin (AVP) and in situ hybridization signals of glutamic acid decarboxylase (GAD)67-mRNA in the suprachiasmatic nucleus (SCN). **a**, **c** AVP immunoreactivity in the SCN, while **b** and **d**, which are adjacent sections to **a** and **c**, respectively, show GAD67-mRNA expression. Note that there are more GAD67-mRNA-positive neurons than AVP-immunoreactive neurons in the SCN. The *dashed lines* delineate the SCN according to AVP immunoreactivity. *Scale bar* 0.5 mm

**Fig. 4:**
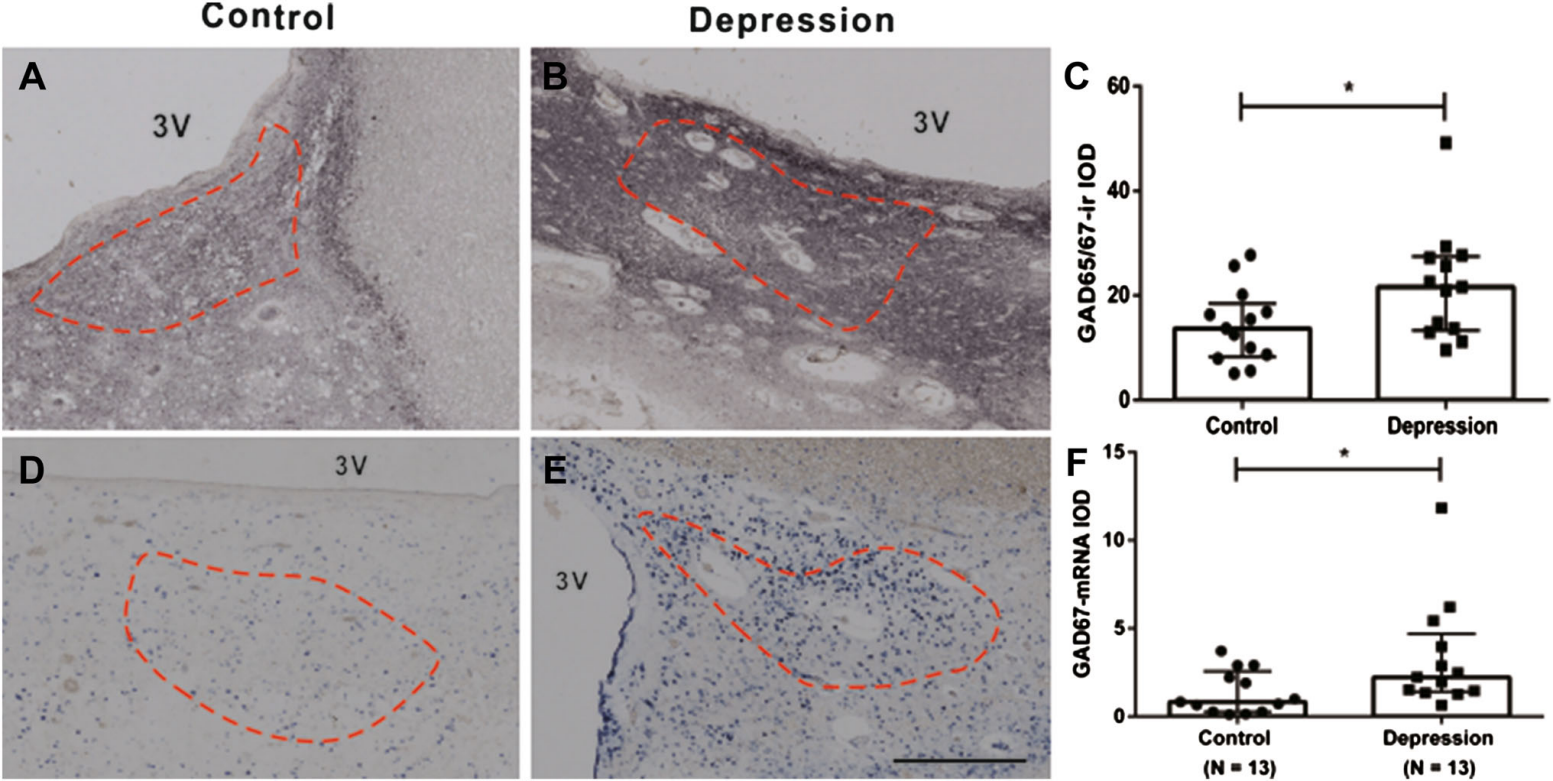
Increased levels of glutamic acid decarboxylase (GAD)65/67-immunoreactivity (ir) and GAD67-mRNA in the suprachiasmatic nucleus (SCN) in depression. Representative GAD65/67-ir and GAD67-mRNA expression in control (**a** and **d**, respectively) and depression patients (**b** and **e**, respectively) in the SCN. The SCN area was *outlined* according to arginine vasopressin staining in an adjacent section (as shown by the *dashed line*, see also Fig. 1), and the outline was transferred to the GAD65/67-staining or GAD67-mRNA in situ hybridization section. Data in **c** and **f** are shown as median with interquartile range. Note the increased GAD65/67-ir and GAD67-mRNA in depression patients. *3V* third ventricle, *IOD* integrated optical density; *P < 0.05. *Scale bar* 0.5 mm

**Fig. 5:**
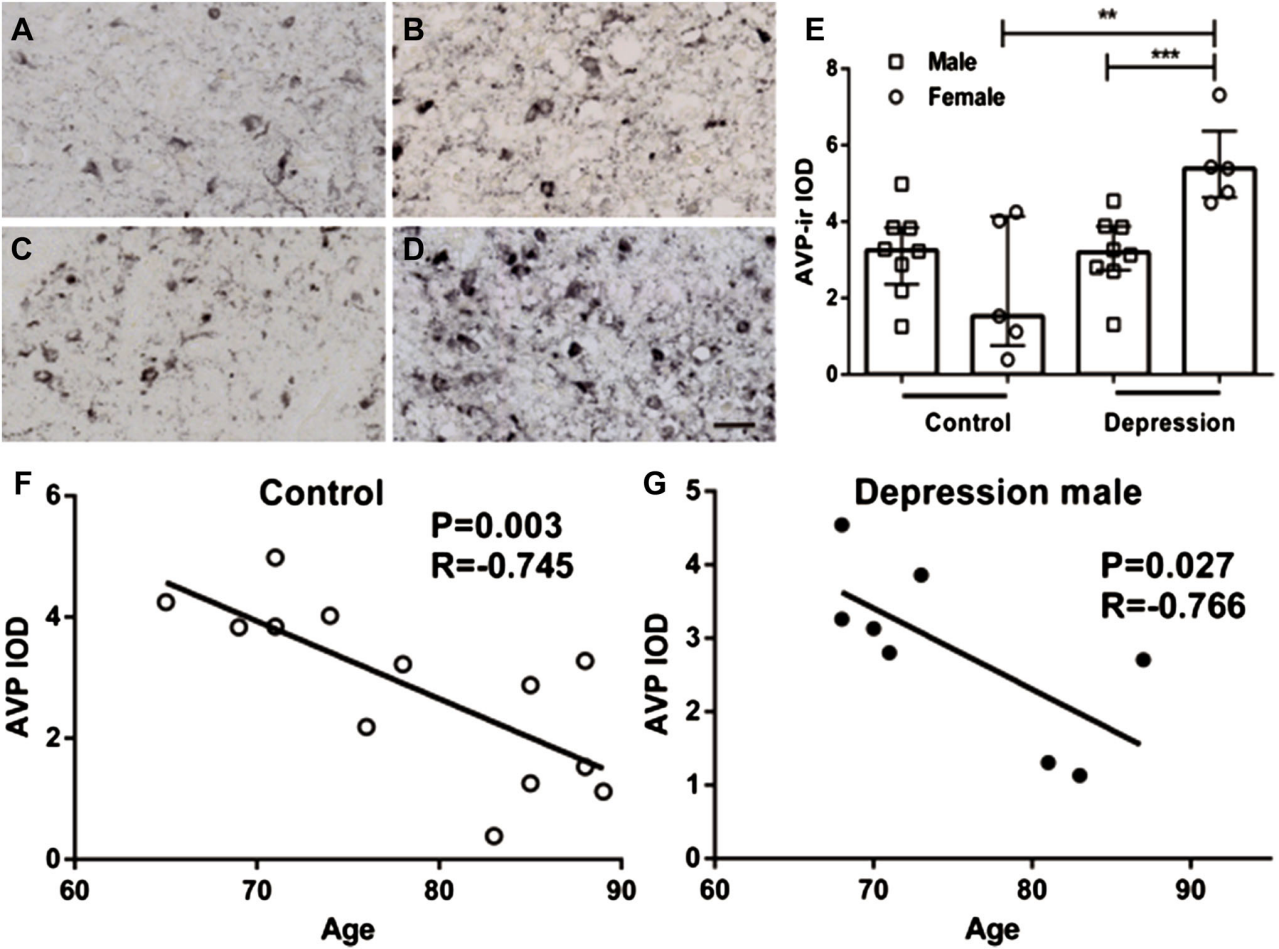
Sex difference in changes of arginine vasopressin (AVP)-immunoreactivity (ir) in the suprachiasmatic nucleus (SCN) in relation to depression or age. **a**–**d** Representative images of AVP-ir of control male (**a**), control female (**b**), depression male (**c**), and depression female (**d**), respectively. Note the increased AVP-ir in depression female patients (**e**). Data are shown as median with interquartile range in **e**. In addition, there was a significant negative correlation between age and SCN AVP-ir in the control group (**f**), which was also present in the male depression group (**g**), but not in the female depression group (figure not shown). *IOD* integrated optical density, **P < 0.01, ***P < 0.005. *Scale bar* 0.05 mm

Arginine vasopressin-stained neurons as well as nerve fibers were found to be widely distributed, not only in the SCN but also in the PVN, SON, and the accessory nuclei (Supplement Figure 1). Figure 3 shows that the majority of the SCN neurons were GABAergic, i.e., that the SCN of these patients contains more GAD-mRNA positive neurons than AVP-staining neurons. GAD65/67-staining and GAD67-mRNA were observed to be widely distributed in the hypothalamus, including in the SCN (Fig. 2). Almost all SCN neurons were surrounded by GAD65/67-ir containing beads, probably terminals, while only few GAD65/67-stained neurons were visible in the SCN (Fig. 2f). In the rostral part of the SCN, a relatively clear boundary of GAD-ir was observed, which was similar to that stained by AVP, while more caudally, the possibility of delineating the SCN by GAD65/67-staining gradually disappeared as the GAD65/67-staining became widely spread throughout the hypothalamus, except for the optic tract (Fig. 2c, d). This was why we used AVP-staining in alternating sections to delineate the GAD65/67-staining in the SCN. The IODs of SCN GAD65/67-ir and GAD67-mRNA were significantly (58 and 169%) higher, respectively, in the depressive samples [21.63 (13.33–27.46) and 2.23 (1.40–4.70), respectively, *n* = 13] compared with control subjects [13.65 (8.29–18.47) and 0.83 (0.27–2.56), respectively, *n* = 13, *P* = 0.044, and *P* = 0.029, respectively, Fig. 4]. In addition, there were a highly significant increase of AVP-ir by 253% in the female depression patients [5.39 (4.63–6.37), *n* = 5] compared with female control subjects [1.53 (0.76–4.14), *n* = 5, *P* = 0.008, Fig. 5a–e], while there was no significant difference between male controls [3.25 (2.36–3.84), *n* = 8] and male depression patients [2.96 (1.66–3.70), *n* = 8, *P* = 0.574, Fig. 5a–e]. Multiple linear regression analysis revealed that there was indeed a clear trend for an association between AVP-ir and sex (*β* = 0.423, *P* = 0.057). Moreover, since the possible sex difference in the expression of SCN AVP-ir in depression was not investigated in one of our group’s previous studies (Zhou et al. 2001), we re-analyzed those data and confirmed the presence of a significantly increased number of AVP-expressing neurons only in females [female control 3422 (1945–4298); female depression 7947 (6362–11,070), *P* = 0.029], while only a trend of increase was present in the male depression group [male control 3534 (2467–6098); male depression 6178 (3832–7276), *P* = 0.073]. Furthermore, in that cohort, we observed a significantly higher ratio of AVP-ir/AVP-mRNA in the depression group [0.78 (0.51–3.24)] compared with the control group (0.30 (0.23–0.51), *P* = 0.002), although the ratio did not show a sex difference. There was a significant negative correlation between AVP-ir and age in the control group (*ρ* = −0.745, *P* = 0.003, *n* = 13), which was also present in the male depression group (*ρ* = −0.766, *P* = 0.027, *n* = 8, Fig. 5f, g) but not in the female depression group (*ρ* = −0.014, *P* = 0.964, *n* = 5). Such a negative correlation between AVP-ir and age was not present in male (*P* = 0.123, *R* = −0.590, *n* = 8) or female (*P* = 0.188, *R* = −0.700, *n* = 5) controls, but it should be noted that the sample sizes of these subgroups were small. SCN GAD65/67-ir or SCN GAD67-mRNA did not show a significant correlation with age, either in the control or in the depression group (all *P* ≥ 0.291).

There were no significant differences in the levels of AVP-ir, GAD65/67-ir, and GAD67-mRNA among the MDD, BD, and control groups (*P* = 0.192, *P* = 0.127, and *P* = 0.074, respectively). Please note that the sample sizes of these subgroups were small. No significant correlation was observed between depression duration and GAD65/67-ir (*ρ* = −0.127, *P* = 0.680), GAD67-mRNA (*ρ* = 0.242, *P* = 0.426), or AVP-ir (*ρ* = 0.030, *P* = 0.922).

## Discussion

In the present study, we found, for the first time, significantly increased GAD67-mRNA and GAD65/67-ir in the SCN, which indicates the presence of increased SCN GABA neurotransmission in depression. In addition, increased SCN AVP-ir was observed only in female patients, not in male patients. This significant sex difference was confirmed in a different cohort after re-analysis of our previously published data (Zhou et al. 2001).

It has been shown in rodents that both SCN GABA and AVP take part in generating circadian activities by interacting with other SCN neuropeptides (Irwin and Allen 2010). A major and novel finding of our present study is that both GAD65/67-ir and GAD67-mRNA were up-regulated in the SCN of depression patients. Based upon the evidence that (1) most human SCN neurons seem to express GABA (Gao and Moore 1996), as was also confirmed by the present study, especially in depressed patients, (2) all of these neurons were surrounded by GAD65/67-ir containing beads, probably terminals, (3) in rodents, GABA may up- or down-regulate SCN function, depending on the photoperiod (Farajnia et al. 2014; Myung et al. 2015), time of the day, and the sub-area of the SCN (DeWoskin et al. 2015; Irwin and Allen 2009; De Jeu and Pennartz 2002; Choi et al. 2008; Albus et al. 2005), (4) tonic release of GABA shifts the molecular clock and affects SCN synchrony (DeWoskin et al. 2015), and (5) SCN GABA may also serve as an efferent signal that conveys timing information to other brain areas and/or regulates the responsiveness of SCN neurons to afferent signals (Trachsel et al. 1996; Buijs et al. 1994), the strong SCN GABA alterations we observed can make a clear contribution to the disrupted biological rhythms in depression. It should be noted that previous studies have found a deficit in cortical GABA levels (Hasler et al. 2007): GABA_A_ receptors (Klumpers et al. 2010) and decreased cortical GABAergic neurons (Rajkowska et al. 2007) in depression. In addition, a significant reduction of GAD-mRNA and GAD-protein was observed in BD (Fatemi et al. 2005; Heckers et al. 2002) and MDD patients (Fatemi et al. 2005; Perry et al. 1977). Several studies have shown that plasma GABA levels remain low after the depression symptoms improved (Petty et al. 1995) or following anti-depressant intake (Petty et al. 1993). However, Bielau et al. (2007) have found increased density of GAD neurons in the orbitofrontal cortex, and in the present study, we have found increased GAD65/67-ir and GAD67-mRNA in the SCN of depression patients. These data indicate, therefore, a presence of brain area-dependent changes of GABAergic systems in depression, which may explain, at least partly, the inconsistent curative effect of using GABAergic agonists as antidepressants (Brambilla et al. 2003).

In rats, SCN AVP was found to increase the firing rates of neurons and the circadian amplitude and to cause phase shifts (Murphy et al. 1998). The significantly increased SCN AVP-ir in female but not male patients (the present study and the paper by Zhou et al. 2001) indicates a stronger functional SCN alteration in female than in male depression patients, which is in agreement with the finding that young moderately depressed women showed higher homeostatic sleep pressure than depressed men (Frey et al. 2012). In addition, the significant negative correlation between SCN-AVP-ir and age in control subjects is in accordance with our previous findings (Swaab et al. 1985). It is a novel finding that this correlation was present in the male but not in the female depression group. These data fit well with the findings that females are more vulnerable to depression than males (Altemus 2006). The SCN molecular clock mechanism is based upon elegant interactive transcription–translation feedback loops for clock genes. AVP is required for the circuit-level organization of the expression of the SCN clock genes (Edwards et al. 2016). Indeed, our group has observed lower levels of AVP-mRNA together with higher levels of AVP-ir in depression in a previous study (Zhou et al. 2001). There seems to be a disbalance in the transcription–translation relationship for SCN AVP in depression, which was confirmed by the present findings following re-analysis of the published data, i.e., there were indeed a significant increase in the ratio of AVP-ir to AVP-mRNA in the depression group compared with controls in that study (Zhou et al. 2001). An important question is whether the alterations in the SCN may not only be the root of circadian changes, but could also be contributing factors to the increased activity of the HPA-axis and so to the symptoms of depression. In nocturnal animals, such as rats, SCN-AVP was found to inhibit hypothalamic HPA activity in the PVN (Kalsbeek et al. 1996), which may imply that a disorder in the SCN in rodents may cause disinhibition of the HPA-axis activity and thus contribute to HPA-axis hyperactivity, leading to depression-like symptoms (Holsboer 2001). However, in a diurnal animal, i.e., the *Arvicanthis ansorgei*, AVP from the SCN was found to have a stimulating effect on the hypothalamic PVN and thus on HPA activity (Kalsbeek et al. 2008). Whether the increased SCN AVP-ir found in the female depression group also leads to a stimulating effect on the HPA activity and thus to more depressive symptoms should be a topic for further studies, since in our previous study (Zhou et al. 2001), we found decreased SCN AVP-mRNA in depression, which prompted our proposal of a reduced production and release of AVP from the SCN. In this respect, it merits attention that recent studies have found that AVP plays a crucial role in the generation of overt circadian rhythms and that diminished AVP signaling in the SCN may cause a loss of coherent circadian rhythms (Li et al. 2009; Yamaguchi et al. 2013; Mieda et al. 2015; Loh et al. 2015). It should also be noted that the hyperactivity of the HPA axis in depression may contribute to the disturbance of the SCN, since we observed that glucocorticoids show an inhibitory effect on AVP mRNA expression in the human SCN (Liu et al. 2006). To the best of our knowledge, there are no data on the effects of CRH or corticosteroids on GABA expression in the SCN, which remains an intriguing topic for future studies.

## Conclusions

A strongly increased SCN GABA was found that may be central to the disrupted clock function in depression. In addition, the functional consequences of the significantly increased SCN AVP-ir in female depression patients for SCN function and its output may be related to the higher vulnerability for depression in women. Taken together, our findings indicate a reduced stimulatory output from the SCN, i.e., there were more inhibitory GABAergic signaling and less excitatory AVP signaling from the SCN to the projection areas in depression, especially in female patients.

## Electronic supplementary material

Below is the link to the electronic supplementary material.

Supplementary material 1 (TIFF 4828 kb)

Supplementary material 2 (DOC 88 kb)

## References

[CR1] Abrahamson E, Leak R, Moore R (2001). The suprachiasmatic nucleus projects to posterior hypothalamic arousal systems. NeuroReport.

[CR2] Albus H, Vansteensel MJ, Michel S, Block GD, Meijer JH (2005). A GABAergic mechanism is necessary for coupling dissociable ventral and dorsal regional oscillators within the circadian clock. Curr Biol.

[CR3] Altemus M (2006). Sex differences in depression and anxiety disorders: potential biological determinants. Horm Behav.

[CR4] Bao AM, Meynen G, Swaab DF (2008). The stress system in depression and neurodegeneration: focus on the human hypothalamus. Brain Res Rev.

[CR5] Belenky MA, Sollars PJ, Mount DB, Alper SL, Yarom Y, Pickard GE (2010). Cell-type specific distribution of chloride transporters in the rat suprachiasmatic nucleus. Neuroscience.

[CR6] Bielau H, Steiner J, Mawrin C, Trubner K, Brisch R, Meyer-Lotz G, Brodhun M, Dobrowolny H, Baumann B, Gos T, Bernstein HG, Bogerts B (2007). Dysregulation of GABAergic neurotransmission in mood disorders: a postmortem study. Ann N Y Acad Sci.

[CR7] Bowers G, Cullinan WE, Herman JP (1998). Region-specific regulation of glutamic acid decarboxylase (GAD) mRNA expression in central stress circuits. J Neurosci.

[CR8] Brambilla P, Perez J, Barale F, Schettini G, Soares JC (2003). GABAergic dysfunction in mood disorders. Mol Psychiatry.

[CR9] Buijs RM, Hou YX, Shinn S, Renaud LP (1994). Ultrastructural evidence for intra- and extranuclear projections of GABAergic neurons of the suprachiasmatic nucleus. J Comp Neurol.

[CR10] Buijs RM, Wortel J, Hou YX (1995). Colocalization of γ-aminobutyric acid with vasopressin, vasoactive intestinal peptide, and somatostatin in the rat suprachiasmatic nucleus. J Comp Neurol.

[CR11] Choi HJ, Lee CJ, Schroeder A, Kim YS, Jung SH, Kim JS, Kim DY, Son EJ, Han HC, Hong SK, Colwell CS, Kim YI (2008). Excitatory actions of GABA in the suprachiasmatic nucleus. J Neurosci.

[CR12] Dai J, Swaab DF, Van der Vliet J, Buijs RM (1998). Postmortem tracing reveals the organization of hypothalamic projections of the suprachiasmatic nucleus in the human brain. J Comp Neurol.

[CR13] De Jeu M, Pennartz C (2002). Circadian modulation of GABA function in the rat suprachiasmatic nucleus: excitatory effects during the night phase. J Neurophysiol.

[CR14] DeWoskin D, Myung J, Belle MD, Piggins HD, Takumi T, Forger DB (2015). Distinct roles for GABA across multiple timescales in mammalian circadian timekeeping. Proc Natl Acad Sci USA.

[CR15] Edwards MD, Brancaccio M, Chesham JE, Maywood ES, Hastings MH (2016). Rhythmic expression of cryptochrome induces the circadian clock of arrhythmic suprachiasmatic nuclei through arginine vasopressin signaling. Proc Natl Acad Sci USA.

[CR16] Farajnia S, van Westering TL, Meijer JH, Michel S (2014). Seasonal induction of GABAergic excitation in the central mammalian clock. Proc Natl Acad Sci USA.

[CR17] Fatemi SH, Stary JM, Earle JA, Araghi-Niknam M, Eagan E (2005). GABAergic dysfunction in schizophrenia and mood disorders as reflected by decreased levels of glutamic acid decarboxylase 65 and 67 kDa and Reelin proteins in cerebellum. Schizophr Res.

[CR18] Frey S, Birchler-Pedross A, Hofstetter M, Brunner P, Gotz T, Munch M, Blatter K, Knoblauch V, Wirz-Justice A, Cajochen C (2012). Young women with major depression live on higher homeostatic sleep pressure than healthy controls. Chronobiol Int.

[CR19] Gao B, Moore RY (1996). Glutamic acid decarboxylase message isoforms in human suprachiasmatic nucleus. J Biol Rhythms.

[CR20] Gao SF, Klomp A, Wu JL, Swaab DF, Bao AM (2013). Reduced GAD(65/67) immunoreactivity in the hypothalamic paraventricular nucleus in depression: a postmortem study. J Affect Disord.

[CR21] Hasler G, van der Veen JW, Tumonis T, Meyers N, Shen J, Drevets WC (2007). Reduced prefrontal glutamate/glutamine and gamma-aminobutyric acid levels in major depression determined using proton magnetic resonance spectroscopy. Arch Gen Psychiatry.

[CR22] Heckers S, Stone D, Walsh J, Shick J, Koul P, Benes FM (2002). Differential hippocampal expression of glutamic acid decarboxylase 65 and 67 messenger RNA in bipolar disorder and schizophrenia. Arch Gen Psychiatry.

[CR23] Holsboer F (2001). Stress, hypercortisolism and corticosteroid receptors in depression: implications for therapy. J Affect Disord.

[CR24] Irwin RP, Allen CN (2009). GABAergic signaling induces divergent neuronal Ca^2+^ responses in the suprachiasmatic nucleus network. Eur J Neurosci.

[CR25] Irwin RP, Allen CN (2010). Neuropeptide-mediated calcium signaling in the suprachiasmatic nucleus network. Eur J Neurosci.

[CR26] Kalsbeek A, van der Vliet J, Buijs RM (1996). Decrease of endogenous vasopressin release necessary for expression of the circadian rise in plasma corticosterone: a reverse microdialysis study. J Neuroendocrinol.

[CR27] Kalsbeek A, Verhagen LA, Schalij I, Foppen E, Saboureau M, Bothorel B, Buijs RM, Pevet P (2008). Opposite actions of hypothalamic vasopressin on circadian corticosterone rhythm in nocturnal versus diurnal species. Eur J Neurosci.

[CR28] Klumpers UM, Veltman DJ, Drent ML, Boellaard R, Comans EF, Meynen G, Lammertsma AA, Hoogendijk WJ (2010). Reduced parahippocampal and lateral temporal GABA_A_-[^11^C]flumazenil binding in major depression: preliminary results. Eur J Nucl Med Mol Imaging.

[CR29] Lewy AJ, Lefler BJ, Emens JS, Bauer VK (2006). The circadian basis of winter depression. Proc Natl Acad Sci USA.

[CR30] Li JD, Burton KJ, Zhang C, Hu SB, Zhou QY (2009). Vasopressin receptor V1a regulates circadian rhythms of locomotor activity and expression of clock-controlled genes in the suprachiasmatic nuclei. Am J Physiol Regul Integr Comp Physiol.

[CR31] Liu RY, Unmehopa UA, Zhou JN, Swaab DF (2006). Glucocorticoids suppress vasopressin gene expression in human suprachiasmatic nucleus. J Steroid Biochem Mol Biol.

[CR32] Loh DH, Kudo T, Colwell CS (2015). Short circuiting the circadian system with a new generation of precision tools. Neuron.

[CR33] Martensson B, Pettersson A, Berglund L, Ekselius L (2015). Bright white light therapy in depression: a critical review of the evidence. J Affect Disord.

[CR34] Mieda M, Ono D, Hasegawa E, Okamoto H, Honma K, Honma S, Sakurai T (2015). Cellular clocks in AVP neurons of the SCN are critical for interneuronal coupling regulating circadian behavior rhythm. Neuron.

[CR35] Moore RY, Speh JC (1993). GABA is the principal neurotransmitter of the circadian system. Neurosci Lett.

[CR36] Moore RY, Speh JC, Leak RK (2002). Suprachiasmatic nucleus organization. Cell Tissue Res.

[CR37] Morris DW, Trivedi MH, Fava M, Wisniewski SR, Balasubramani GK, Khan AY, Jain S, Rush AJ (2009). Diurnal mood variation in outpatients with major depressive disorder. Depression Anxiety.

[CR38] Murphy HM, Wideman CH, Nadzam GR (1998). The role of vasopressin in modulating circadian rhythm responses to phase shifts. Peptides.

[CR39] Myung J, Hong S, DeWoskin D, De Schutter E, Forger DB, Takumi T (2015). GABA-mediated repulsive coupling between circadian clock neurons in the SCN encodes seasonal time. Proc Natl Acad Sci USA.

[CR40] Neylan TC (1995). Treatment of sleep disturbances in depressed patients. J Clin Psychiatry.

[CR41] Perry EK, Gibson PH, Blessed G, Perry RH, Tomlinson BE (1977). Neurotransmitter enzyme abnormalities in senile dementia. Choline acetyltransferase and glutamic acid decarboxylase activities in necropsy brain tissue. J Neurol Sci.

[CR42] Petty F, Steinberg J, Kramer GL, Fulton M, Moeller FG (1993). Desipramine does not alter plasma GABA in patients with major depression. J Affect Disord.

[CR43] Petty F, Kramer GL, Fulton M, Davis L, Rush AJ (1995). Stability of plasma GABA at four-year follow-up in patients with primary unipolar depression. Biol Psychiatry.

[CR44] Rajkowska G, O’Dwyer G, Teleki Z, Stockmeier CA, Miguel-Hidalgo JJ (2007). GABAergic neurons immunoreactive for calcium binding proteins are reduced in the prefrontal cortex in major depression. Neuropsychopharmacology.

[CR45] Rybakowski JK, Dmitrzak-Weglarz M, Dembinska-Krajewska D, Hauser J, Akiskal KK, Akiskal HH (2014). Polymorphism of circadian clock genes and temperamental dimensions of the TEMPS-A in bipolar disorder. J Affect Disord.

[CR46] Swaab DF, Fliers E, Partiman TS (1985). The suprachiasmatic nucleus of the human brain in relation to sex, age and senile dementia. Brain Res.

[CR47] Trachsel L, Dodt HU, Zieglgansberger W (1996). The intrinsic optical signal evoked by chiasm stimulation in the rat suprachiasmatic nuclei exhibits GABAergic day–night variation. Eur J Neurosci.

[CR48] Wu YH, Zhou JN, Balesar R, Unmehopa U, Bao A, Jockers R, Van Heerikhuize J, Swaab DF (2006). Distribution of MT1 melatonin receptor immunoreactivity in the human hypothalamus and pituitary gland: colocalization of MT1 with vasopressin, oxytocin, and corticotropin-releasing hormone. J Comp Neurol.

[CR49] Yamada N, Martin-Iverson MT, Daimon K, Tsujimoto T, Takahashi S (1995). Clinical and chronobiological effects of light therapy on nonseasonal affective disorders. Biol Psychiatry.

[CR50] Yamaguchi Y, Suzuki T, Mizoro Y, Kori H, Okada K, Chen Y, Fustin JM, Yamazaki F, Mizuguchi N, Zhang J, Dong X, Tsujimoto G, Okuno Y, Doi M, Okamura H (2013). Mice genetically deficient in vasopressin V1a and V1b receptors are resistant to jet lag. Science (New York, NY).

[CR51] Zhou JN, Riemersma RF, Unmehopa UA, Hoogendijk WJ, van Heerikhuize JJ, Hofman MA, Swaab DF (2001). Alterations in arginine vasopressin neurons in the suprachiasmatic nucleus in depression. Arch Gen Psychiatry.

[CR52] Zhu QB, Unmehopa U, Bossers K, Hu YT, Verwer R, Balesar R, Zhao J, Bao AM, Swaab D (2016). MicroRNA-132 and early growth response-1 in nucleus basalis of Meynert during the course of Alzheimer’s disease. Brain.

