# Supplementary material for: Increased glutamic acid decarboxylase expression in the hypothalamic suprachiasmatic nucleus in depression
Source: Brain Struct Funct. 2017 Jun 12;222(9):4079–88. doi: 10.1007/s00429-017-1442-y (PMC5686266; doi:10.1007/s00429-017-1442-y)
Supplement: Supplementary file 2 — Supplementary material 2 (DOC 88 kb) [file 429_2017_1442_MOESM2_ESM.doc]

**Supplement**

**Increased glutamic acid decarboxylase expression in the hypothalamic suprachiasmatic nucleus in depression**

Xueyan Wu • Rawien Balesar • Jing Lu • Sahar Farajnia • Qiongbin Zhu • Manli Huang • Aimin Bao • Dick F. Swaab

**Table** Clinico-pathological information of subjects

| NBB | Group | Sex | Age (y) | PMD (hr:min) | FT (d) | CTD | MOD | CSF pH | BW (g) | Braak stage | Medication in the past | Suicide attempt | Cause of death |
| --- | --- | --- | --- | --- | --- | --- | --- | --- | --- | --- | --- | --- | --- |
|  |  |  |  |  |  |  |  |  |  |  |  |  |  |
| 94-032 | MDD | M | 71 | 16:15 | 38 | 16:15 | 2 | ND | 975 | 0 | ZUC, BZD, MAOI, clomipramine | Yes | Probably broncho-pnemonia, next to cerebral ischaemia |
| 94-017 | MDD | F | 72 | 22:00 | 39 | 19:00 | 1 | ND | 1287 | 1 | TeCA, BZD, prednison | No | Bronchopneumonia, mesothelioma |
| 12-097 | MDD | F | 73 | 5:45 | 61 | 15:30 | 9 | 6.70 | 1205 | 3 | TeCA | No | Heart failure, legal euthanasia |
| 11-058^a^ | MDD | M | 83 | 10:40 | 57 | 05:00 | 7 | 6.50 | 1200 | 2 | TCA, TeCA, Hal, Pipamperon, SSRI | No | PTSS with depression, acute heart failure |
| 08-076 | MDD | F | 91 | 5:20 | 36 | 09:05 | 8 | 6.53 | 1163 | 3 | Hal, SSRI, BZD, levothyroxine | No | Cachexia and dehydration by pneumonia and renal insufficiency |
| 08-031 | MDD | F | 93 | 4:20 | 51 | 04:55 | 3 | 6.80 | 1023 | 2 | SSRI, BZD | Yes | Pneumonia |
| 02-014 | BD | M | 68 | 12:00 | 30 | 00:00 | 2 | 6.64 | 1414 | 1 | Li, Hal, ZUC, MAOI | No | Subdural hematoma after fall |
| 99-118 | BD | M | 68 | 5:55 | 33 | 23:15 | 10 | 6.82 | 1174 | 1 | Li, SSRI | No | Cardiac ischemia |
| 00-111 | BD | M | 70 | 4:50 | 43 | 02:45 | 10 | 6.26 | 1442 | 1 | Li, ZUC, valproate, BZD,ECT | No | Cardiac arrest, ileus due to intestinal haemorrhage |
| 00-088^b^ | BD | M | 73 | 5:15 | 36 | 09:30 | 7 | 6.38 | 1145 | 2 | Li, BZD, SSRI, Hal, ECT, MAOI, Methylphfenidaat | No | Cachexia, dehydration |
| 98-010 | BD | F | 75 | 4:00 | 38 | 20:45 | 1 | ND | 1123 | 1 | TeCA, TCA | No | Acute abdomen secondary to a perforation of stomach / intestines due to NSAIDs |
| 12-048 | BD | M | 81 | 6:40 | 60 | 20:00 | 5 | 6.70 | 1283 | 1 | Li, prednisolon | No | Legal euthanasia |
| 12-110 | BD | M | 87 | 3:15 | 53 | 23:00 | 10 | 6.39 | 1285 | 3 | BZD, valproate | No | CVA, pneumania |
| Median | - | - | 78 | 5:45 | 39 | 15:30 |  | 6.58 | 1205 | 1 | - | - | - |
| 97-042 | CTR | F | 65 | 12:50 | 28 | 02:00 | 4 | 6.94 | 910 | 1 | None | - | Cardiac arrest, pneumonia, pulmonary oedema |
| 99-101 | CTR | M | 69 | 19:15 | 41 | 03:30 | 8 | 6.40 | 1337 | 1 | None | - | Pneumonia, small inarction in brainstem |
| 92-049 | CTR | M | 71 | 5:40 | 32 | 0:00 | 4 | 7.40 | 1250 | 0 | None | - | Found death |
| 08-032 | CTR | M | 71 | 8:55 | 70 | 03:15 | 3 | 6.64 | 1520 | 2 | Mo | - | Pancreas carcinoma, rectum carcinoma with hepatic metastases |
| 98-104 | CTR | F | 74 | 7:25 | 31 | 09:50 | 7 | 6.95 | 1167 | 2 | BZD | - | Necrosis of the intestins secondary to thrombosis |
| 06-028 | CTR | M | 76 | 19:35 | 27 | 20:00 | 4 | 6.50 | 1494 | 3 | None | - | Prostate carcinoma, cardiac arrest |
| 94-039 | CTR | M | 78 | 53 | 88 | 12:00 | 1 | ND | 1354 | 0 | None | - | Myocardial infarction |
| 99-116 | CTR | M | 78 | 4:20 | 43 | 16:45 | 9 | ND | 1300 | 0 | None | - | Pancreats carcinoma |
| 00-022 | CTR | F | 83 | 7:45 | 34 | 21:00 | 2 | 6.52 | 1072 | 2 | Digoxin, methimazole | - | Acute myocardial infarction |
| 09-075 | CTR | M | 88 | 7:00 | 44 | 02:25 | 10 | 6.76 | 1230 | 3 | Salbutamol, prednisolone, | - | Cachexia and dehydration by rectum carinoma and prostste carcinoma |
| 08-105 | CTR | F | 89 | 3:52 | 58 | 0:10 | 12 | 7.30 | 1258 | 3 | prednisolone | - | Pneumonia |
| 99-044 | CTR | F | 88 | 5:55 | 34 | 07:00 | 4 | 6.05 | 1115 | 1 | BZD | No | Cardiac arrest |
| 90-080 | CTR | M | 85 | 4:55 | 28 | 11:50 | 11 | 6.34 | 1035 | 3 | None | No | Myocardial infraction |
| 00-007 | CTR | M | 85 | 15:10 | 35 | 0,938 | 1 | 6,85 | 1328 | 2 | None | No | Myocardial infraction |
| Median | - | - | 73 | 7:45 | 34 | 7:00 |  | 6.64 | 1250 | 2 | - | - | - |
| P-value | - | - | 0.619 | 0.361 | 0.223 | 0.186 | 0.571 | 0.771 | 0.650 | 0.801 | - | - | - |

**Note:** BD, bipolar disorder; Braak stage, progression of pathological changes for Alzheimer’s disease according to Braak et al., 1991; BW, brain weight; BZD, benzodiazepine; CSF, cerebrospinal fluid; CTD, clock time at death; CTR, control; CVA, cerebrovascular accident; ECT, electshock treatment; F, female; FT(d), fixation time in days; F, female; Hal, haloperidol; Li, lithium; M, male; MAOI, monoamine oxidase inhibitor; MDD, major depressive disorder; Mo, morphine; MOD, month of death; NBB, Netherlands Brain Bank; ND, no data; None, no medication; NSAIDs, nonsteroidal anti-inflammatory drugs; PMD, postmortem delay; PTSS, posttraumatische stressstoornis; SSRI, selective serotonin reuptake inhibitor; TCA, tricyclic antidepressant; TeCA, tetracyclic antidepressants; ZUC, zuclopenthixol. a: Patient also be diagnosis with post traumatic stress syndrome. b: Patient also be diagnosis with old cerebrovascular accident mildly demended.

**Methods**

**Immunocytochemistry of GAD65/67-ir and AVP-ir in the SCN staining protocols**

For AVP staining, the sections were deparaffinized and rehydrated using xylene and decreasing grades of ethanol. After rinsing in 0.05 M Tris-buffered saline (TBS) (pH 7.6), the sections were placed in 0.05 M Tris-HCl buffer (pH 7.6) and microwaved for antigen retrieval at 720 W for 10 minutes. The sections were then incubated overnight at 4 °C with monoclonal mouse anti-AVP (D-7) diluted in supermix (1xTBS, 0.5%Triton, 0.25 % gelatin, pH 7.6). The next day the sections were incubated for 60 minutes at RT with biotinylated anti-mouse (Vector Laboratories, Burlingame, Calif., USA) 1: 400 diluted in supermix, and then incubated in ABC elite kit (Vector Labs, Burlingame, Calif., USA, 1: 800) for 60 minutes. Finally, the sections were incubated for 20 minutes in DAB-Ni substrate solution (TBS containing 0.5 mg/ml 3,3-diaminobenzidine (Sigma), 0.01% H2O2 and 0.2% nickel ammonium sulfate, pH 7.6).

The procedure for GAD65/67 staining was similar to that for AVP-staining, except that the sections were microwaved for antigen retrieval in sodium citrate buffer (pH 6.0). The dilution of the first antibody was 1:1200, the 2^nd^ antibody was anti-rabbit (Vector Laboratories, Burlingame, Calif., USA) and the sections were finally developed in DAB-Ni for 10 minutes.

**In situ hybridization for GAD67-mRNA in the SCN staining protocols**

The slides were deparaffinized in xylene baths and rehydrated by a series of ethanol dilutions. Sections were pretreated by microwave at 800 W in 0.01 M sodium citrate buffer (pH 6.0), digested with 0.2N HCL followed by proteinase-K digestion. The sections were then hybridized overnight. After that, the slides were washed in pre-heated saline sodium and then incubated in anti-fluorescein-alkaline phosphatase conjugated antibody for 3 hours at RT. The slides were then incubated with nitro-blue tetrazolium chloride 5-Bromo-4-Chloro-3'-Indolylphosphatase p-Toluidine salt and levamisole - in the dark - to develop the dark-blue precipitate. Sections were then washed in distilled water, rinsed in methanol for 5 minutes and coverslipped with glycerine.

**Data collection and calculation of quantitative image analyses**

A computer program was designed to mask and extract the AVP-ir, GAD65/67-ir and GAD-mRNA positive signal structures. The threshold for the positive signal was set at twice the optical density (OD) of the background. The computer determined the OD of the pixels and percentage surface area covered by the signal (area mask). The integrated optical density (IOD) was calculated by multiplying the OD with the masked area corrected for background. For each subject, the total IOD was calculated as the final parameter for the total amount of AVP-ir, GAD65/67-ir or GAD67-mRNA by a conversion program based upon multiplication of the separate IOD by sample frequency of the sections, as was described previously .

**Reference**

Goldstone AP, Unmehopa UA, Bloom SR, Swaab DF (2002) Hypothalamic NPY and agouti-related protein are increased in human illness but not in Prader-Willi syndrome and other obese subjects. The Journal of clinical endocrinology and metabolism 87 (2):927-937. doi:10.1210/jcem.87.2.8230

**Figure legends**

**Supplement Figure 1:** Specific immunocytochemical staining in the hypothalamus. Arginine vasopressin (AVP; A, C and E) and oxytocin (OXT; B, D and F) staining were shown in the suprachiasmatic nucleus (SCN, A-B), supraoptic nucleus (SON, C-D) and the accessory nuclei, or the ‘islands’ (E-F), which are located between the paraventricular nucleus (PVN) and the SON. B, D and F are adjacent sections to A, C and E, respectively. Please note the AVP-staining by antibody D-7 (A), while OXT-staining by antibody AІ -28 (B) was lacking in the SCN. In addition, AVP-staining was present in the large majority of the large SON neurons (C), while a few different, smaller OXT-stained cells were present in the dorsal cap of the SON (D). In the islands, AVP-staining (E) and OXT-staining (F) were also present in different cells and fibers. These findings indicate that anti-AVP and anti-OXT antibodies do not cross react with the protocols used in the present study. 3V, the third ventricle. Insertions: higher magnification in the same anatomical position in adjacent sections for AVP- and OXT-staining. Scale bar = 0.25 mm in A-F, and = 0.15 mm in insertions.
